# Pet Owners’ Perceptions of COVID-19, Zoonotic Disease, and Veterinary Medicine: The Impact of Demographic Characteristics

**DOI:** 10.3390/vetsci9050195

**Published:** 2022-04-19

**Authors:** Lauren Powell, Tyler M. Lavender, Chelsea L. Reinhard, Brittany Watson

**Affiliations:** School of Veterinary Medicine, University of Pennsylvania, Philadelphia, PA 19104, USA; tml@vet.upenn.edu (T.M.L.); creinh@vet.upenn.edu (C.L.R.); brittawa@vet.upenn.edu (B.W.)

**Keywords:** access to care, COVID-19, one health, human–animal interactions, public health, SARS-CoV-2, zoonoses

## Abstract

This study aimed to investigate the impact of sociodemographic characteristics on pet owners’ concern about the transmission of zoonotic disease and SARS-CoV-2, and to describe owners’ perceptions of veterinarians and physicians as resources for zoonoses information. Between September and October 2020, 1154 individuals completed an online survey via Qualtrics. Binary logistic regression models were used to examine the associations between owner demographics and perceptions of zoonoses and SARS-CoV-2. Most participants were minimally concerned about their pets contracting or transmitting zoonotic diseases or SARS-CoV-2, although perceptions of risk differed based on age, race, and education. Older participants were typically less concerned about the transmission of zoonotic diseases and SARS-CoV-2. Considering where participants obtained information about zoonoses, pet owners were more likely to contact their veterinarian for advice (43%) than their physician (17%). However, 17% of pet owners struggled to access veterinary care, and 51% said their access to veterinary care had become more difficult during the COVID-19 pandemic. Our findings highlight a need for further education about zoonoses and SARS-CoV-2, and suggest veterinarians may play a key role in these communications. The results also emphasize the need to address access to care issues in veterinary medicine.

## 1. Introduction

COVID-19 has emerged as one of the most impactful infectious diseases in recent history. Declared a global pandemic by the World Health Organization (WHO) on 11 March 2020 [1], COVID-19 has led to more than 5 million reported human fatalities globally [2]. While the scale of the COVID-19 pandemic has been unprecedented, the looming threat of zoonotic diseases, such as COVID-19, has been recognized by the public health community for some time [3,4]. Research published two decades ago showed that 60% of infectious diseases and 75% of emerging diseases were zoonotic, and that zoonotic pathogens were two times more likely to emerge than non-zoonotic pathogens [5]. Opportunities for zoonotic transmission of disease have also increased due to human population growth, increased food demand, changes in land use, international travel and trade, the widespread use of antimicrobial drugs, and the reduced proximity between animals and humans, which is particularly relevant for pet owners [4,6,7].

Although COVID-19 is spread almost exclusively through human–human transmission, experimental studies have demonstrated human–animal and animal–animal transmission of SARS-CoV-2, the causative agent of COVID-19, in several species, such as cats, farmed mink, and non-human primates [8,9]. Considering the global popularity of pet ownership, including 57% of US households [10], the possible transmission of SARS-CoV-2 between humans and companion animals is particularly concerning. While there appears to be negligible transmission of SARS-CoV-2 from companion animals to humans, evidence suggests that between 20 and 67% of cats and dogs become infected with SARS-CoV-2 following exposure to a COVID-positive owner [11,12,13,14]. Cats are more susceptible to symptomatic infection, which can include mild to moderate respiratory and gastrointestinal disease [8]. Cat–cat transmission has also been recorded through both direct contact and aerosols [15,16,17]. At both the time of data collection and the time of publication, the Centers for Disease Control and Prevention (CDC) and the American Veterinary Medical Association (AVMA) did not recommend routine testing of cats and dogs for SARS-CoV-2 and considered the risk of zoonotic transmission from domestic animals to humans to be negligible [18]. However, other species, such as ferrets, are susceptible to SARS-CoV-2 infection and transmission [19], and possible reports of animal–human transmission have led to widespread culling in some countries [20].

Preventative health behaviors can drastically reduce the transmission of disease during outbreaks, and individual’s knowledge and perceptions of disease have a significant impact on their willingness to adopt such behaviors [21,22,23]. For example, increased knowledge of COVID-19 has been associated with greater risk perception and increased adoption of health-preventative behaviors, such as social distancing and mask wearing [21]. A similar positive association exists between individuals’ knowledge of other zoonoses, such as Lyme disease and Hendra virus, and their risk perception and use of preventative behaviors [24,25]. Perceptions and awareness of zoonoses also vary with demographic variables. Characteristics, such as being female, married, and having a post-graduate education, have been associated with increased knowledge of COVID-19 and increased adoption of preventative behaviors [26]. Awareness of rabies also tends to be higher among females and those with higher educational attainment [23]. Understanding the impact of demographic factors on perceptions of zoonoses is crucial to identify target groups for intervention to improve health literacy.

An individual’s access to medical and/or veterinary care may also affect their awareness and perceptions of zoonoses, as both physicians and veterinarians can represent valuable source of information regarding zoonotic diseases. Research suggests that veterinarians tend to diagnose zoonotic disease more frequently than physicians and are typically more confident providing advice about zoonoses prevention and risk mitigation [27,28,29]. Data also suggest that physicians believe veterinarians should be involved in managing zoonoses [28], and more than one-fifth of physicians have referred their patients to a veterinarian regarding zoonoses [27]. However, collaboration between physicians and veterinarians in zoonoses management is typically limited [27,28,29]. This study aimed to identify possible associations between pet owners’ sociodemographic factors and their perceptions of risk pertaining to the zoonotic transmission of disease with pets, and specifically the zoonotic transmission of SARS-CoV-2. A secondary aim was to describe pet owners’ access to veterinary care and their perceptions of veterinarians and physicians as resources for zoonoses information.

## 2. Materials and Methods

### 2.1. Participants

Participants were recruited to complete the survey online between 10 September 2020 and 16 October 2020. This study was advertised through social media postings and online mailing lists to relevant industry groups, such as the Association for Shelter Veterinarians listserv and the Association of Veterinary Medical College’s Primary Care Veterinary Educators listserv. The survey was also shared in the University of Pennsylvania School of Veterinary Medicine alumni newsletter. To be eligible, participants had to be aged 18 years or older. This study was reviewed by the University of Pennsylvania Institutional Review Board and determined to be exempt (protocol number 843856).

### 2.2. Survey

The survey was available through Qualtrics (Qualtrics, Provo, UT, USA) and all responses were recorded anonymously. The full survey included 41 questions (Supplementary Materials). Participants were first asked about their sociodemographic characteristics and pet ownership history, including their pet-related expenses and barriers to accessing veterinary care. Participants were also shown two open-ended questions concerning their barriers to accessing veterinary care, which were coded by the first author (LP) using an open-coding method. The next block of questions focused on participants’ prior knowledge of zoonotic diseases and their level of concern regarding zoonotic disease transmission between themselves and their pets on a five-point Likert scale. We then provided the participants with a statement about the possible, albeit low, risk of zoonotic transmission of SARS-CoV-2, and asked questions about their level of concern regarding COVID-19 and their concern about the risk of zoonotic transmission of SARS-CoV-2 with their pets using five-point Likert scales. Participants then completed several questions about their willingness to consult their physician or veterinarian for advice about zoonotic diseases. Finally, we provided participants with the AVMA’s current stance on testing pets for SARS-CoV-2 (i.e., that routine testing for SARS-CoV-2 in animals is not recommended [18]), before asking whether they were likely to get their pets tested for SARS-CoV-2 if the current recommendations were to change, and also their comfort level with having a SARS-CoV-2-positive pet in their home, again using five-point Likert scales.

### 2.3. Statistical Analysis

Descriptive statistics were calculated to show the demographic characteristics of the sample and pet owners’ level of concern about zoonoses and COVID-19. Significant outliers were removed from two variables considering the amount of money participants were willing to pay for veterinary care, including values ≥$1,000,000 for emergency veterinary care (*n* = 34) and values ≥$10,000 for SARS-CoV-2 testing of pets (*n* = 2). Kendall’s tau-b was used to assess the level of correlation between respondent concern for contracting COVID-19 and concern about their pets contracting or transmitting SARS-CoV-2, their willingness to get their pets tested for SARS-CoV-2, the amount of money they were willing to pay for a SARS-CoV-2 test and their level of comfort with having a SARS-CoV-2-positive pet in the home. Binary logistic regression models were then used to examine the associations between demographic characteristics (age, gender, annual household income, education level, race, household structure and region within the United States) and (1) access to veterinary care (difficult/not difficult); (2) change in access during the COVID-19 pandemic (more difficult/no change or easier); (3) awareness of zoonotic disease (had not heard term ‘zoonotic disease’/heard term ‘zoonotic disease’, and did not know meaning of zoonotic disease/knew meaning of zoonotic disease); (4) perceptions of zoonotic disease (concerned/not concerned); (5) perceptions of COVID-19 (concerned/not concerned); (6) willingness to get pet tested for SARS-CoV-2 (likely/neutral or unlikely); and 7) comfort having a SARS-CoV-2-positive pet in the home (uncomfortable/neutral or comfortable). Due to the small number of cases in some categories, race was collapsed to only include Caucasians, African Americans, and Asians, although we were still unable to include race in the model about concern for contracting COVID-19. Mann–Whitney U tests were conducted to examine the relationship between respondents’ awareness of zoonotic disease (had heard term, knew definition) and the likelihood of asking physicians or veterinarians about zoonotic diseases. All statistical analyses were performed in IBM SPSS Statistics. *p* < 0.05 was considered statistically significant.

## 3. Results

### 3.1. Demographics

The survey was initiated by 1397 individuals, although 242 did not complete the survey and one respondent did not provide consent, leaving a final sample of 1154. The descriptive characteristics of the sample are provided in Table 1. Almost all of the participants were pet owners (*n* = 1148, 99.5%), including 77.6% who owned at least one dog (median one dog, range one-14), 48.5% who owned at least one cat (median two cats, range one-14) and 15.5% participants who owned other types of pets, such as horses, rabbits, hamsters and guinea pigs (*n* = 179). Approximately 20% of the participants had pet insurance (*n* = 252, 21.8%) and the vast majority reported their pets were up to date on vaccinations (*n* = 1068, 93.0%).

### 3.2. Perceptions of Zoonotic Disease and COVID-19

Prior to completing the survey, half of the participants had heard the term “zoonotic disease” (*n* = 579, 50.2%) and 53.9% indicated they knew what a zoonotic disease was (*n* = 622). Respondents with an undergraduate or postgraduate degree were significantly more likely to have heard the term ‘zoonotic disease’ and know the meaning compared with respondents with a high school education or less (Table 2). Individuals who lived in the Midwest and Western United States were more likely to have heard the term zoonotic disease, and respondents from the Western United States were also more likely to know the definition of a zoonotic disease compared with respondents from the Northeast. Conversely, older participants and those with higher levels of income were less likely to have heard the term ‘zoonotic disease’ or know the definition of a zoonotic disease.

**Figure 1:**
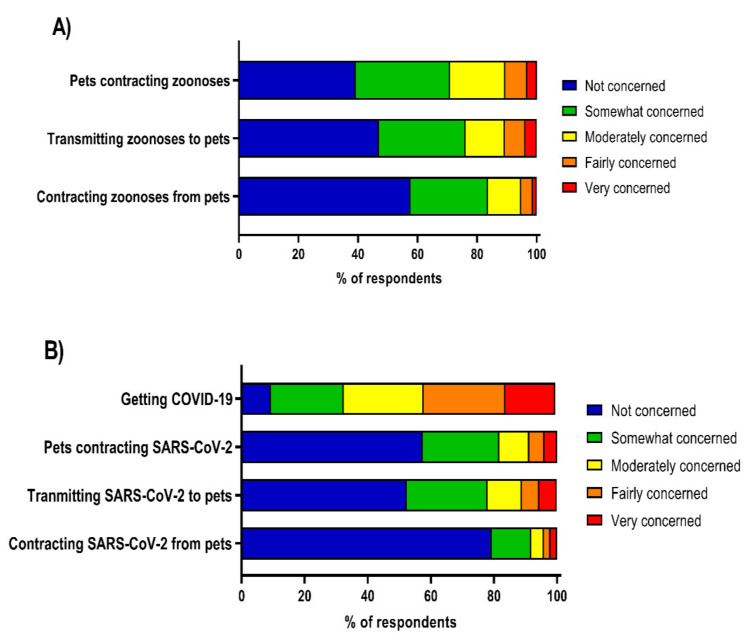
(**A**) Respondent concern about transmission of zoonotic diseases; and (**B**) respondent concern about COVID-19 and zoonotic transmission of SARS-CoV-2.

In general, most participants were not concerned or only somewhat concerned about their pets contracting or transmitting zoonotic diseases or SARS-CoV-2, whereas most participants were moderately or fairly concerned about contracting COVID-19 themselves (Figure 1). Participants’ level of concern for contracting COVID-19 was strongly associated with their concern for their pets contracting SARS-CoV-2 (*τ*b = 0.32, *p* < 0.001) and moderately correlated with their concern about getting SARS-CoV-2 from their pets (*τ*b = 0.23, *p* < 0.001).

Almost half of the participants were extremely (*n* = 134, 11.6%) or somewhat uncomfortable (*n* = 385, 33.4%) at the prospect of having a SARS-CoV-2-positive pet in their home. Approximately one-quarter were neither comfortable nor uncomfortable (*n* = 317, 27.5%), and a similar proportion were either somewhat (*n* = 174, 15.1%) or extremely comfortable (*n* = 144, 12.5%) with having a SARS-CoV-2-positive pet in their home. Concern about contracting COVID-19 was weakly, negatively associated with comfort having a SARS-CoV-2-positive pet in the home (*τ*b = −0.19, *p* < 0.001).

### 3.3. Demographic Characteristics and Perceptions of Zoonotic Disease and Transmission of SARS-CoV-2

Table 3 and Table 4 show the associations between demographic characteristics and participants’ perceptions of the risk of zoonotic transmission of disease and zoonotic transmission of SARS-CoV-2, respectively. When including all demographic factors in the models, we found age, race and education were associated with participants’ concern about zoonotic diseases. Participants aged 30 years or older were less likely to be concerned about their pets contracting or transmitting zoonotic diseases, and participants aged over 40 were less likely to be concerned about contracting zoonotic disease from their pets than those under the age of 30. African Americans were 52% less likely to be concerned about their pets contracting zoonotic diseases than Caucasians (OR 0.48, 95% CI 0.24–0.96). Participants with an undergraduate degree were also less likely to be concerned about contracting zoonotic diseases from their pets than those with a high school education or less (OR 0.67, 95% CI 0.46–0.99).

Participants’ concern about contracting COVID-19 was associated with age and education. Participants aged 40–49 years and 50–59 years were significantly less likely to indicate they were concerned about contracting COVID-19 than participants aged 18–29 (OR 0.41, 95% CI 0.21–0.78, and OR 0.43, 95% CI 0.23–0.79, respectively), while individuals with a postgraduate education were almost four times more likely to be concerned about contracting COVID-19 than those with a high school education or less (OR 3.81, 95% CI 1.84–7.86). Concern about zoonotic transmission of SARS-CoV-2 was associated with education level, but not gender, race, ethnicity, annual household income, household structure, or region within the United States. Although individuals with a postgraduate degree were much more likely to be concerned about contracting COVID-19 than participants with a high school diploma or less, the opposite relationship was found between education level and concern about zoonotic transmission of SARS-CoV-2. Participants with an undergraduate or postgraduate university degree were 59% and 71% less likely to be concerned about their pets contracting SARS-CoV-2 compared with individuals with a high school education or less (OR 0.41, 95% CI 0.26–0.63, and OR 0.29, 95% CI 0.18–0.46). Considering transmitting SARS-CoV-2 to their pets, participants with an undergraduate degree were 65% less likely to report concern (OR 0.35, 95% CI 0.11–0.54) and those with a postgraduate degree were 64% less likely to be concerned than participants with a high school diploma or less (OR 0.36, 95% CI 0.22–0.57). Participants’ comfort with having a SARS-CoV-2-positive pet in the home was associated with age, in that participants in the 50–59 age bracket were 59% more likely to be comfortable having a SARS-CoV-2-positive pet in their home than participants aged 18–29 years (OR 1.59, 95% CI 1.07–2.36).

### 3.4. SARS-CoV-2 Testing in Pets

Considering SARS-CoV-2 testing in pets, 14.3% (*n* = 165) of participants were extremely unlikely, 16.8% (*n* = 194) were somewhat unlikely and 16.1% (*n* = 186) were neither likely nor unlikely to get their pets tested for SARS-CoV-2 if the current guidelines were to change and the test was available for free. Approximately half the participants were somewhat (*n* = 324, 28.1%) or extremely likely (*n* = 285, 24.7%) to get their pets tested for SARS-CoV-2 if the test was free. When asked if they would pay to get their pets tested for SARS-CoV-2, more participants indicated they were extremely (*n* = 333, 28.9%) or somewhat unlikely (*n* = 342, 28.1%) to pay to get their pets tested for SARS-CoV-2, while a similar proportion were neither likely nor unlikely (16.9%, *n* = 195). Approximately one-third of participants were somewhat (23.2%, *n* = 268) or extremely likely (8.2%, *n* = 95) to pay for their pets to be tested for SARS-CoV-2. Participants said they would be willing to spend a mean $78.58 to get their pet tested for SARS-CoV-2 (SD $286.38, median $40.00, IQR $20.00–$75.00).

**Figure 2:**
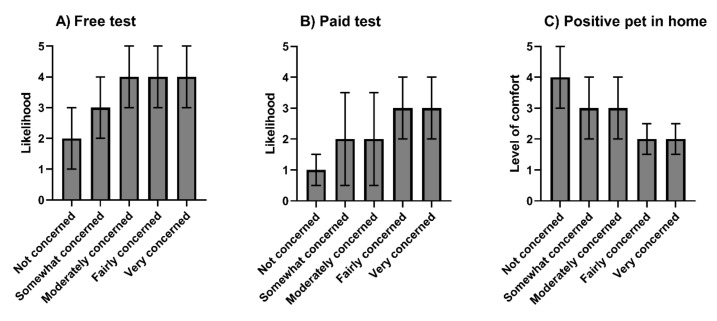
Concern about contracting COVID-19 relative to: (**A**) the likelihood of getting a pet tested for SARS-CoV-2 in the test were free; (**B**) the likelihood of getting a pet tested for SARS-CoV-2 if there was a fee; and (**C**) the level of comfort with having a SARS-CoV-2-positive pet in the home. Data are shown as the median ± IQR.

We found a moderate, positive correlation between participants’ concern for contracting COVID-19 and the likelihood of getting their pets tested for SARS-CoV-2, irrespective of whether the test was free (*τ*b = 0.26, *p* < 0.001) or had a cost associated (*τ*b = 0.23, *p* < 0.001, Figure 2). The strength of the correlation was comparable between dog owners (free test *τ*b = 0.26, paid test *τ*b = 0.22) and cat owners (free test *τ*b = 0.25, paid test *τ*b = 0.27). There was also a weak, positive trend between concern for contracting COVID-19 and the amount of money that participants were willing to pay for a SARS-CoV-2 test for their pet (*τ*b = 0.14, *p* < 0.001).

Table 5 shows the associations between participants’ demographic characteristics and their willingness to get their pets tested for SARS-CoV-2 as a binary variable (likely/not likely), including all demographic variables and adjusting for concern about contracting COVID-19. Age was the sole demographic factor associated with participants’ willingness to get their pets tested for SARS-CoV-2 if the test was free. Participants in the 30–39 and 50–59 age brackets were significantly less likely to report they would get their pet tested for SARS-CoV-2 if the test were free than those aged 18–29 years. Considering paid SARS-CoV-2 tests for pets, annual household income was significantly associated with the likelihood of getting pets tested for SARS-CoV-2. Participants who earned $30,000–$49,999, $100,000–$349,999 or >$350,000 were significantly more likely to report they would get their pets tested for SARS-CoV-2 than those who earned <$30,000. In both models, participants’ level of concern for contracting COVID-19 was more powerful than any demographic factor in predicting the likelihood of getting their pets tested for SARS-CoV-2.

### 3.5. Perceptions of Physicians and Veterinarians as Resources for Zoonotic Disease Information

**Figure 3:**
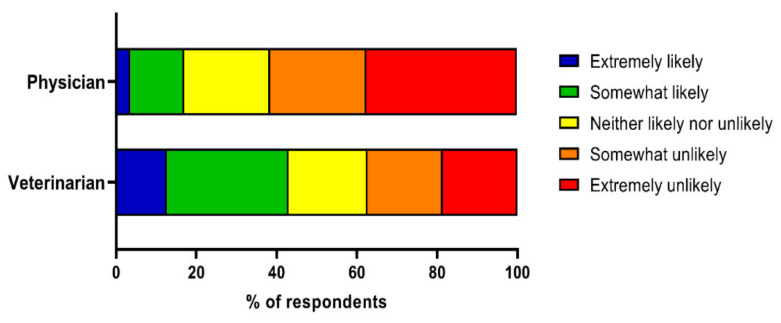
Likelihood of participants contacting their physician and veterinarian for information about zoonotic diseases.

Figure 3 displays participants’ responses to the question “how likely are you to ask a physician/veterinarian about zoonotic diseases?” Considerably more participants said they were somewhat or extremely likely to contact their veterinarian for information about zoonoses than their physician, including 43.0% of participants and 17.0%, respectively. Additionally, 37.9% of participants said their veterinarian had talked to them about zoonotic diseases in the past (*n* = 437), whereas only 10.4% of participants said their physician had ever spoken to them about zoonotic disease (*n* = 120). Mann–Whitney U tests showed respondents who had heard the term ‘zoonotic disease’ and knew the meaning were more likely to ask their veterinarian about zoonotic diseases than those who had not heard the term or did not know the meaning (*U* = 209,289, *Z* = 5.77, *p* < 0.001, and *U* = 205,529, *Z* = 5.29, *p* < 0.001, respectively). There were no significant differences between respondents who had heard the term or knew the meaning of zoonotic disease and the likelihood of contacting physicians about zoonotic disease (*p* ≥ 0.19).

### 3.6. Access to Veterinary Care

Most participants reported they could access veterinary care somewhat easily (*n* = 306, 26.5%) or extremely easily (*n* = 422, 36.6%), although 19.6% of participants found it neither easy nor difficult to access veterinary care (*n* = 226), 16.1% reported it was somewhat difficult (*n* = 186) and 1.2% found it extremely difficult to access veterinary care (*n* = 14). Among those participants who said veterinary care was somewhat or extremely difficult to access, 38% said cost prevented them from accessing veterinary care (*n* = 76) and 16.0% said the distance to the clinic or inadequate transportation prevented them from accessing care (*n* = 32). No participants reported language or communication barriers to care prior to the COVID-19 pandemic. In the open-text responses, four participants said they had difficulty accessing veterinary care due to their animal’s behavioral problems and two participants said time constraints prevented them from accessing care. Many other owners reported COVID-related access to care issues which are described further below.

Most participants indicated veterinary care was somewhat (*n* = 472, 40.9%) or very affordable (*n* = 160, 13.9%) at the time of survey completion. Yet, 17.9% of participants reported veterinary care was neither unaffordable nor affordable (*n* = 207), 23.3% reported it was somewhat unaffordable (*n* = 269) and 4.0% reported veterinary care was extremely unaffordable (*n* = 46). Participants indicated they would be willing to spend a mean $4,881.29 on veterinary care in an emergency (SD $9671.52, median $2500, IQR $1000–$5000).

Household income and race were the only demographic characteristics that predicted ease of access to veterinary care. Participants that had an annual household income of $100,000–$350,000 were two times more likely to report veterinary care was not difficult to access compared with individuals earning less than $30,000 (OR 1.94, 95% CI 1.09–3.45). We also found African American participants were 65% less likely to report veterinary care was not difficult to access compared with Caucasian participants (OR 0.35, 95% CI 0.17–0.72).

### 3.7. Changes in Access to Veterinary Care during the COVID-19 Pandemic

Almost half the sample said their access to veterinary care did not change during the COVID-19 pandemic (*n* = 549, 47.6%), although 44.5% of participants found veterinary care somewhat more difficult to access (*n* = 514) and 6.2% found it much more difficult to access during the pandemic (*n* = 72). A smaller proportion of participants found access to veterinary care was somewhat easier (*n* = 15, 1.3%) or much easier during the COVID-19 pandemic (*n* = 4, 0.3%).

The most common reason that owners faced increased difficulties accessing veterinary care during the COVID-19 pandemic was due to limited availability of veterinary appointments, including long wait times, limited services and/or the provision of emergency/sick appointments only (42.7%, *n* = 250). Of those who faced increased challenges accessing care during the COVID-19 pandemic, one-quarter had difficulties accessing care due to the cost of services or their ability to pay (*n* = 141, 24.1%), and 21.7% faced challenges due to the use of curbside care and their inability to accompany their pets during the veterinary appointment (*n* = 127). A further 11.3% of owners faced difficulties due to inadequate transportation/the distance to the clinic (*n* = 66), 6.7% struggled with reduced operating hours during the COVID-19 pandemic (*n* = 39), and 4.6% had difficulties with communication or language barriers, often due to curbside care and their inability to communicate directly with the veterinarian (*n* = 27). A few participants had difficulty accessing care due to their concerns about contracting COVID-19 (*n* = 23, 3.9%), their animal’s behavior (*n* = 15, 2.6%) and time constraints (*n* = 14, 2.4%). Of the 19 participants that found access to care easier during the pandemic, six indicated they preferred curbside care, due to reduced waiting, increased parking availability, and not having to leave the car, and two owners enjoyed the use of telemedicine.

A binary logistic regression model found no significant associations between demographic characteristics, including age, gender, race, education, household income and region within the US, and the likelihood of participants experiencing increased difficulty accessing veterinary care during the COVID-19 pandemic (χ^2^(19) = 16.14, *p =* 0.65).

## 4. Discussion

The primary goal of this study was to investigate the impact of sociodemographic factors on pet owners’ perceptions of zoonotic diseases and zoonotic transmission of SARS-CoV-2. Although zoonoses present a significant risk to public health [5], only half of the participants in this study had heard the term “zoonotic disease” or knew the definition of a zoonotic disease, and most were minimally concerned about the transmission of zoonoses between themselves and their pets. Similarly, in a small sample of Australian pet owners, only one in 10 owners said they were concerned about contracting a disease from their pet and almost one-quarter said they never considered the possibility [30]. A number of zoonotic diseases are endemic in the United States and globally that can pose a risk to both humans and pets [31,32]. The relatively low levels of awareness of zoonoses highlights a crucial knowledge gap and a need for further education among pet owners. For example, over one million people are estimated to be infected with *Toxoplasma gondii* each year in the United States [33] which can occur through contact with cat feces, such as through cleaning a litter box, in addition to other pathways, such as consumption of undercooked meat, contaminated water or vegetables [31]. Rabies, which is largely spread through dogs, is estimated to kill approximately 60,000 people around the world each year [34] and zoonotic multidrug-resistant bacteria have been found in the companion animal population which amplifies the risk to human health [35,36,37].

Pet owners’ perceptions of zoonoses were consistently associated with age. Owners over the age of 30, and particularly those over the age of 60, were less likely to have heard the term ‘zoonotic disease’ or know the meaning, to be concerned about contracting zoonotic diseases from their pets, transmitting zoonotic diseases to their pets, or their pets contracting zoonotic disease compared with owners under the age of 30. Health literacy, i.e., the ability to acquire, understand and apply health information, is typically lower among older adults and minority populations which could explain the reduced level of concern about zoonoses among older respondents [38]. African American participants were also less likely to be concerned about their pets contracting zoonotic diseases than Caucasians, although the relationship did not hold true when pet owners considered the risk of transmitting or contracting zoonotic diseases themselves. There were also no differences in awareness of zoonotic disease (i.e., having previously heard the term ‘zoonotic disease’ or knowing the meaning) based on race. Pet-keeping practices may vary between African American respondents and Caucasians which could influence perceptions of risk pertaining to zoonotic disease. For example, previous research has shown that African American pet owners are less likely to allow their pets to sleep in their bed than White pet owners [39]. So, African Americans in this study may have perceived a lower risk of zoonotic transmission from their pets and, therefore, may have been less concerned about their pets contracting zoonotic disease. We also found pet owners with an undergraduate degree were significantly less concerned about contracting zoonotic diseases from their pets than owners with a high school education. Health literacy is typically higher with higher levels of education [38], so it is not clear why owners with an undergraduate degree were less concerned about contracting zoonotic disease. There were no significant differences in access to veterinary care based on education. However, increased educational attainment has been linked to increased access to human healthcare [40], so it is possible that university-educated owners were less concerned about contracting zoonoses due to their increased access to medical resources.

Like the transmission of zoonoses, most pet owners were not concerned or only somewhat concerned about the risk of zoonotic transmission of SARS-CoV-2, although many were moderately concerned about contracting COVID-19 themselves. These findings are somewhat at odds with those of a previous survey that found approximately 50% of veterinarians reported their clients were concerned about potential of SARS-CoV-2 transmission with their pets [41]. In Italy, research has also shown that 28% of dog owners altered their behavior during the early stages of the COVID-19 pandemic by cleaning their dogs following walks and ceasing participation in sporting activities [42]. Education was the key demographic variable that impacted owners’ perception of zoonotic transmission of SARS-CoV-2. Owners with an undergraduate or postgraduate degree were less likely to be concerned about contracting SARS-CoV-2 from their pets, transmitting SARS-CoV-2 to their pets, or their pets contracting SARS-CoV-2. At the time of survey completion, very few companion animals had tested positive for SARS-CoV-2 [43], and major health and veterinary organizations, such as the CDC, WHO, and the AVMA, had released statements describing the low risk of transmission of SARS-CoV-2 from pets to humans [44]. University-educated pet owners may have been aware of such reports and perhaps had a better understanding of the primary routes of SARS-CoV-2 transmission, leading to reduced concern about the risk of zoonotic transmission. The impact of education on owners’ perceptions of zoonotic transmission of SARS-CoV-2 also suggests that educational outreach to the community could be a key step towards improving zoonoses health literacy among underserved populations.

Despite the relatively low levels of concern about zoonotic transmission of SARS-CoV-2, almost half of the sample were somewhat or extremely uncomfortable at the idea of having a SARS-CoV-2-positive pet in their home. Participants with higher levels of concern about contracting COVID-19 reported decreased comfort having a SARS-CoV-2-positive pet in the home. We also found that pet owners in the 50–59-year age bracket were more comfortable having a SARS-CoV-2-positive pet in their home than younger pet owners, likely due to their decreased concern about contracting COVID-19. This finding was somewhat surprising considering the increased risk of health complications associated with COVID-19 among older adults [45]. Previous research has shown other demographic characteristics, such as political party affiliation, also significantly impact individual’s concern for contracting COVID-19 [46,47]. Such characteristics were not captured by this survey but may contribute to the lower levels of concern for contracting COVID-19 among older respondents.

Half of the pet owners in this study indicated they would get their pets tested for SARS-CoV-2 if it was recommended and the test was available for free, and approximately 30% were willing to pay for their pets to be tested for SARS-CoV-2, up to a median of $40. The relatively low proportion of participants who would get their pets tested for SARS-CoV-2, even if testing was recommended by the AVMA, is potentially worrisome for human and animal health. The transmission of SARS-CoV-2 among pets could have obvious detrimental impacts on the health status of companion animals, but it could also lead to increased cases among humans if pets were to serve as a disease reservoir [48,49]. As described above, data available at the time of data collection indicated that the risk of SARS-CoV-2 transmission among companion animals was exceedingly low [44] and the AVMA did not recommend routine testing of pets. It is possible that pet owners were less inclined to get their pets tested for SARS-CoV-2 because of these data.

Participants’ level of concern for contracting COVID-19 was again a key predictor of willingness to get pets tested for SARS-CoV-2, regardless of cost, and was considerably more impactful than any demographic characteristics. Given owners’ concern for contracting COVID-19 was positively correlated with their concern about contracting SARS-CoV-2 from their pets, our findings suggest that owners were more willing to get their pets tested if they were concerned about their personal health outcomes and the risk of zoonotic transmission from their pets. Age was negatively associated with the likelihood of owners reporting they would be willing to get their pets tested for SARS-CoV-2 for free, which may be attributable to the generally lower levels of concern about zoonotic disease and COVID-19 among older individuals in this sample. Household income was also positively associated with the likelihood of owners reporting they would be willing to pay for a SARS-CoV-2 test for their pet, which is logical and supports previous research that showed the likelihood of visiting a veterinarian increases with owner income [50,51]. Although further research is needed, our findings suggest that interventions targeted towards older age groups may help to improve zoonoses health literacy and the adoption of health behaviors to minimize the risk of zoonotic transmission between owners and pets.

Mirroring previous findings from veterinarians and physicians [27,28,29], we found more pet owners said their veterinarian had discussed zoonoses with them compared with physicians (38% compared with 10%). A greater proportion of owners also said they would consult their veterinarian about zoonoses than those who would consult their physician, which also reflects previous studies [30,52,53]. Individuals who were aware of zoonoses (had heard the term or knew the definition) were also more likely to consult their veterinarian about zoonotic disease than respondents who were not aware of zoonotic diseases. Interestingly, there were no significant differences in the likelihood of respondents asking their physician about zoonotic disease based on their awareness of zoonoses. Veterinarians clearly represent a trusted source of information about zoonoses for many pet owners which speaks to the importance of adopting a One Health approach with collaboration between physicians and veterinarians in the management of zoonoses [27]. Veterinarians might also play a key role in communications regarding risks for SARS-CoV-2 given owners see them as a trusted source on zoonoses.

The above findings emphasize the need to address access to care issues in veterinary medicine. A report from the Access to Veterinary Care Coalition has shown that 23% of pet owners in the United States face difficulties accessing preventative veterinary care, primarily due to financial restraints [54]. Here, we found 17% of pet owners reported veterinary care was difficult to access and 27% of participants said veterinary care was somewhat or extremely unaffordable. Cost has been repeatedly listed as a key barrier to veterinary care among underserved populations [55,56], so it is not surprising that participants with higher incomes of $100,000–$350,000 were two times more likely to have no difficulties accessing veterinary care compared with lower-income pet owners (<$30,000). Previous research has also found that with increasing household income, the proportion of owners who visit the veterinarian regularly and access preventive care, including rabies vaccination, also increases [57]. Transportation/distance to the veterinary clinic was another significant barrier for pet owners in this study. In many undeserved areas, veterinary services are sparce leading to what some have termed “veterinary deserts” or “care deserts” in which owners face intermittent or non-existent access to veterinary care [58]. Such a lack of services may explain, at least in part, the significantly higher rates of difficulty accessing veterinary care among African American pet owners. Veterinarian-client communication and culture/language differences are also common barriers to veterinary care [56] which are likely to disproportionately affect people of color, given the veterinary workforce in the United States is comprised of 90% White veterinarians [59]. No pet owners in this study said that language or communication barriers prevented them from accessing care prior to the COVID-19 pandemic.

Access to veterinary care became increasingly difficult during the pandemic for half of the pet owners in this study. Veterinary clinics often implemented operational changes to meet social distancing requirements, such as reducing appointment availability and providing emergency/sick appointments only [60]. The reduced availability of veterinarians was the most common barrier for owners. Many also said the use of curbside care and their inability to accompany their pets during the appointment was a significant source of stress for themselves and their pets, and a barrier to care. Although owners were not unanimous in their perceptions of curbside care, the use of telehealth services which also increased during the pandemic [41], may have reduced barriers to care in cases where pet owners were uncomfortable being separated from their pets.

Demographic factors did not affect the likelihood of pet owners experiencing increased difficulty accessing veterinary care during the COVID-19 pandemic, suggesting that the aforementioned barriers related to veterinarian availability and the use of curbside care affected many pet owners, irrespective of socioeconomic factors. Other research has found the COVID-19 pandemic led to an increase in the number of unemployed pet owners utilizing low- or no-cost veterinary services [61] and that barriers to veterinary care were compounded for low-income pet owners [62]. For example, pet owners that struggled with transport prior to the pandemic often faced increased difficulties during the pandemic due to the use of shared transport and the increased risk of COVID-19 infection [62]. Similarly, pet owners with disabilities faced increased challenges accessing veterinary care due to exacerbated financial challenges, transportation difficulties and the use of curbside services [63].

This study has provided preliminary insights into pet owners’ perceptions of risk about zoonotic disease transmission between themselves and their pets. However, the findings must be considered in light of several limitations. Firstly, we used convenience sampling, so the sample has an overrepresentation of White, highly educated females from the Northeastern United States. The sample also reported high vaccination rates among pets, which suggests they were a group of pet owners who attend the veterinarian regularly. While the skewed sample does limit the generalizability of our findings, the high levels of educational achievement in the sample further emphasizes the need for increased awareness of the risks of zoonoses among pet owners. It would be particularly interesting to see a comparable study investigating perceptions of zoonoses among underserved pet owners as their access to veterinary care is often hindered. We did not consider respondent’s political beliefs or party affiliations which have been shown to impact perceptions of COVID-19 in the United States and may have contributed to some of the observed differences between demographic groups, particularly the unusual negative relationship between concern for contracting COVID-19 and age [46,47]. Participants’ responses about their concern for zoonotic transmission of SARS-CoV-2 may also have been influenced by the AVMA position statement in the survey that described the limited evidence for zoonotic transmission of SARS-CoV-2 among companion animals. We felt an ethical obligation to include this information to avoid causing undue concern among pet owners and to reduce potential impacts on animal welfare, particularly when considering the occurrence of mass culling events of animals due to fears of zoonotic transmission of SARS-CoV-2 [20]. Another limitation arises from the fact that participants were asked to report their hypothetical intentions and concern about zoonoses, COVID-19 and SARS-CoV-2 testing for pets, rather than their actual behaviors. There is often a disconnect between people’s intentions and their true behaviors [64], so further research is needed to confirm our findings.

## 5. Conclusions

Through this study, we found most owners were not concerned or minimally concerned about the risk of zoonotic transmission of disease and SARS-CoV-2 with their pets. Older pet owners, in particular, were less concerned about transmission of zoonoses. Many pet owners perceived their veterinarians as a source of information regarding zoonoses, more so than physicians, which highlights the need for a collaborative One Health approach in the management of zoonoses. The reliance on veterinarians for information pertaining to zoonoses also reinforces the importance of improving access to veterinary care for all pet owners.

## Figures and Tables

**Table 1:** Demographic characteristics of the sample.

Demographic Characteristics	*n*	%
Gender		
Male	96	8.3
Female	1041	90.2
Non-binary	7	0.6
Prefer not to answer	10	0.9
Age		
18–29	361	31.3
30–39	220	19.1
40–49	173	15.0
50–59	217	18.8
60+	183	15.9
Ethnicity		
Hispanic/Latino/Spanish	46	4.0
Not Hispanic	1108	96.0
Race ^a^		
American Indian/Alaskan Native	6	0.5
Asian	29	2.5
Black/African American	39	3.4
Native Hawaiian/Pacific Islander	2	0.2
White	1055	91.4
Other/Prefer not to answer	36	3.1
Annual household income ^b^		
Less than $30,000	132	11.4
$30,000–$49,999	158	13.7
$50,000–$99,999	356	30.8
$100,000–$350,000	460	39.8
More than $350,000	48	4.2
Education		
High school diploma or less	156	13.5
Associate degree/Undergraduate university degree	584	50.6
Postgraduate university degree	414	35.9
Region		
Northeast	980	84.9
Midwest	23	2.0
South	105	9.1
West	43	3.7
Number of people in house		
≤2	715	62.0
3–4	377	32.7
≥5	62	5.4

^a^ Could select multiple races; ^b^ participants were asked to report income for 2019.

**Table 2:** Logistic regression models showing the associations between demographic characteristics and awareness of zoonotic disease.

	Heard the Term ‘Zoonotic Disease’		Knew the Meaning of ‘Zoonotic Disease’	
Demographic Characteristics	OR (95% CI)	*p*	OR (95% CI)	*p*
Sex ^a^	1.32 (0.84–2.06)	0.23	1.23 (0.79–1.91)	0.36
Age				
18–29	Reference		Reference	
30–39	0.63 (0.43–0.93)	0.02 *	0.69 (0.47–1.01)	0.06
40–49	0.57 (0.38–0.86)	0.01 *	0.65 (0.43–0.98)	0.04 *
50–59	0.47 (0.32–0.69)	<0.001 *	0.48 (0.32–0.70)	<0.001 *
60+	0.47 (0.32–0.71)	<0.001 *	0.47 (0.32–0.70)	<0.001 *
Race				
Caucasian	Reference		Reference	
African American	1.32 (0.54–2.39)	0.74	1.26 (0.59–2.67)	0.56
Asian	0.70 (0.30–1.67)	0.42	0.89 (0.37–2.11)	0.79
Ethnicity				
Hispanic/Latino/Spanish	0.76 (0.35–1.64)	0.48	1.01 (0.47–2.18)	0.98
Education				
High school or less	Reference		Reference	
Undergraduate	2.13 (1.40–3.24)	<0.001 *	1.75 (1.17–2.61)	0.01 *
Postgraduate	3.29 (2.10–5.15)	<0.001 *	2.40 (1.56–3.68)	<0.001 *
Household income				
<$30,000	Reference		Reference	
$30,000–$49,999	0.43 (0.25–0.73)	0.002 *	0.40 (0.23–0.69)	0.001 *
$50,000–$99,999	0.38 (0.24–0.62)	<0.001 *	0.40 (0.25–0.65)	<0.001 *
$100,000–$349,999	0.45 (0.28–0.73)	0.001 *	0.47 (0.29–0.77)	0.002 *
>$350,000	0.39 (0.18–0.83)	0.01 *	0.46 (0.21–0.98)	0.04 *
Number of people in household				
1–2 people	Reference		Reference	
3–4 people	0.92 (0.70–1.22)	0.56	0.87 (0.66–1.15)	0.33
5+ people	0.77 (0.44–1.36)	0.37	0.96 (0.55–1.67)	0.87
US region				
Northeast	Reference		Reference	
Midwest	4.03 (1.32–12.37)	0.02 *	2.67 (0.95–7.52)	0.06
South	1.52 (0.97–2.37)	0.07	1.55 (0.99–2.43)	0.06
West	3.60 (1.63–7.95)	0.002 *	3.65 (1.61–8.26)	0.002 *

OR (95% CI) shows the odds ratio and 95% confidence intervals. * Denotes statistical significance (*p* < 0.05); ^a^ Males coded as the reference category.

**Table 3:** Logistic regression models describing the associations between demographic characteristics and perceptions of zoonotic disease.

	Pets Contracting Zoonotic Disease		Transmitting Zoonotic Disease to Pets		Contracting Zoonotic Disease from Pets	
Demographic Characteristics	OR (95% CI)	*p*	OR (95% CI)	*p*	OR (95% CI)	*p*
Sex ^a^	0.91 (0.59–1.43)	0.69	1.17 (0.76–1.80)	0.48	1.00 (0.64–1.55)	0.99
Age						
18–29	Reference		Reference		Reference	
30–39	0.62 (0.42–0.91)	0.02 *	0.66 (0.46–0.96)	0.03 *	0.69 (0.48–1.00)	0.05
40–49	0.64 (0.42–0.96)	0.03 *	0.56 (0.38–0.84)	0.01 *	0.58 (0.38–0.87)	0.01 *
50–59	0.50 (0.34–0.73)	<0.001 *	0.59 (0.41–0.86)	0.01 *	0.64 (0.44–0.93)	0.02 *
60+	0.49 (0.33–0.73)	<0.001 *	0.51 (0.35–0.75)	0.001 *	0.59 (0.40–0.88)	0.01 *
Race						
Caucasian	Reference		Reference		Reference	
African American	0.48 (0.24–0.96)	0.04 *	0.79 (0.39–1.58)	0.50	0.83 (0.41–1.68)	0.60
Asian	1.60 (0.61–4.15)	0.34	1.25 (0.53–2.99)	0.61	2.03 (0.85–4.86)	0.11
Ethnicity						
Hispanic/Latino/Spanish	0.59 (0.28–1.24)	0.16	0.77 (0.37–1.61)	0.49	1.06 (0.51–2.22)	0.87
Education						
High school or less	Reference		Reference		Reference	
Undergraduate	0.70 (0.47–1.04)	0.08	0.75 (0.51–1.11)	0.15	0.67 (0.46–0.99)	0.04 *
Postgraduate	0.77 (0.50–1.18)	0.23	0.74 (0.49–1.12)	0.15	0.70 (0.46–1.05)	0.08
Household income						
<$30,000	Reference		Reference		Reference	
$30,000–$49,999	1.01 (0.60–1.70)	0.97	1.15 (0.70–1.91)	0.58	1.16 (0.71–1.91)	0.55
$50,000–$99,999	0.79 (0.50–1.25)	0.32	0.84 (0.54–1.30)	0.43	0.87 (0.56–1.35)	0.54
$100,000–$349,999	1.05 (0.66–1.67)	0.85	1.02 (0.65–1.59)	0.95	0.90 (0.58–1.40)	0.64
>$350,000	0.89 (0.42–1.87)	0.75	0.79 (0.38–1.64)	0.53	0.67 (0.32–1.43)	0.30
Number of people in household						
1–2 people	Reference		Reference		Reference	
3–4 people	0.86 (0.65–1.13)	0.28	0.91 (0.69–1.19)	0.48	1.11 (0.84–1.46)	0.46
5+ people	0.81 (0.46–1.41)	0.45	0.90 (0.52–1.56)	0.72	1.21 (0.70–2.10)	0.49
US region						
Northeast	Reference		Reference		Reference	
Midwest	1.02 (0.41–2.52)	0.97	1.12 (0.46–2.73)	0.80	1.27 (0.52–3.07)	0.60
South	0.97 (0.63–1.50)	0.90	0.74 (0.48–1.12)	0.15	0.81 (0.53–1.25)	0.35
West	0.84 (0.42–1.67)	0.62	0.85 (0.43–1.68)	0.64	0.92 (0.46–1.84)	0.81

OR (95% CI) shows the odds ratio and 95% confidence intervals. * Denotes statistical significance (*p* < 0.05); ^a^ Males coded as the reference category.

**Table 4:** Logistic regression models describing the associations between demographic characteristics and perceptions of zoonotic transmission of SARS-CoV-2.

Demographic Characteristics	Contracting COVID-19		Pets Contracting SARS-CoV-2 ^a^		Transmitting SARS-CoV-2 to Pets ^a^		Contracting SARS-CoV-2 from Pets ^a^		Comfort Having SARS-CoV-2+ Pet in Home ^a^	
	OR (95% CI)	*p*	OR (95% CI)	*p*	OR (95% CI)	*p*	OR (95% CI)	*p*	OR (95% CI)	*p*
Sex ^b^	1.67 (0.88–3.19)	0.12	0.75 (0.47–1.20)	0.23	0.91 (0.57–1.46)	0.69	0.72 (0.43–1.23)	0.23	1.19 (0.76–1.86)	0.46
Age										
18–29	Reference		Reference		Reference		Reference		Reference	
30–39	0.95 (0.46–1.95)	0.88	0.97 (0.65–1.44)	0.87	0.84 (0.57–1.25)	0.40	1.05 (0.65–1.69)	0.85	1.25 (0.85–1.83)	0.26
40–49	0.41 (0.21–0.78)	0.01 *	1.06 (0.68–1.66)	0.78	0.92 (0.60–1.42)	0.70	1.08 (0.63–1.86)	0.77	1.03 (0.68–1.56)	0.90
50–59	0.43 (0.23–0.79)	0.01 *	1.17 (0.78–1.76)	0.45	0.91 (0.61–1.37)	0.65	1.28 (0.79–2.07)	0.32	1.59 (1.07–2.36)	0.02 *
60+	0.68 (0.34–1.33)	0.26	1.06 (0.70–1.60)	0.79	0.78 (0.52–1.18)	0.24	1.28 (0.78–2.08)	0.33	0.86 (0.58–1.28)	0.46
Race ^c^										
Caucasian			Reference		Reference		Reference		Reference	
African American			1.31 (0.62–2.78)	0.48	1.48 (0.71–3.11)	0.30	1.80 (0.78–4.15)	0.17	0.55 (0.27–1.13)	0.10
Asian			1.09 (0.44–2.66)	0.84	1.47 (0.60–3.62)	0.40	0.99 (0.35–2.84)	0.99	1.19 (0.50–2.82)	0.69
Ethnicity										
Hispanic/Latino/Spanish	1.03 (0.35–3.03)	0.96	0.46 (0.19–1.07)	0.07	1.15 (0.52–2.52)	0.73	0.87 (0.33–2.26)	0.77	2.01 (0.91–4.47)	0.09
Education										
High school or less	Reference		Reference		Reference		Reference		Reference	
Undergraduate	1.11 (0.64–1.92)	0.70	0.41 (0.26–0.63)	<0.001 *	0.35 (0.11–0.54)	<0.001 *	0.54 (0.34–0.87)	0.01 *	1.16 (0.77–1.73)	0.48
Postgraduate	3.81 (1.84–7.86)	<0.001 *	0.29 (0.18–0.46)	<0.001 *	0.36 (0.22–0.57)	<0.001 *	0.50 (0.30–0.82)	0.01 *	0.98 (0.64–1.51)	0.93
Household income										
<$30,000	Reference		Reference		Reference		Reference		Reference	
$30,000–$49,999	0.91 (0.42–1.98)	0.81	1.02 (0.63–1.86)	0.77	1.20 (0.70–2.05)	0.51	1.73 (0.89–3.39)	0.11	1.03 (0.62–1.74)	0.90
$50,000–$99,999	1.10 (0.55–2.21)	0.79	1.21 (0.73–1.88)	0.52	1.08 (0.68–1.74)	0.74	1.53 (0.84–2.81)	0.17	0.97 (0.62–1.53)	0.90
$100,000–$349,999	1.30 (0.63–2.70)	0.48	1.12 (0.68–1.78)	0.71	1.10 (0.68–1.77)	0.70	1.26 (0.68–2.35)	0.46	0.79 (0.50–1.26)	0.32
>$350,000	2.41 (0.50–11.63)	0.27	1.51 (0.70–3.29)	0.30	1.25 (0.58–2.70)	0.58	1.41 (0.56–3.58)	0.47	0.57 (0.27–1.22)	0.15
Number of people in household										
1–2 people	Reference		Reference		Reference		Reference		Reference	
3–4 people	1.16 (0.72–1.85)	0.54	0.84 (0.63–1.13)	0.25	0.91 (0.68–1.22)	0.52	1.02 (0.72–1.44)	0.93	0.98 (0.74–1.30)	0.91
5+ people	0.86 (0.36–2.06)	0.74	0.82 (0.45–1.50)	0.52	0.84 (0.46–1.50)	0.55	1.22 (0.61–2.43)	0.58	0.76 (0.43–1.34)	0.34
US region										
Northeast	Reference		Reference		Reference		Reference		Reference	
Midwest	1.68 (0.22–13.05)	0.62	0.61 (0.23–1.59)	0.31	0.75 (0.30–1.88)	0.53	0.89 (0.29–2.76)	0.84	2.26 (0.84–6.06)	0.11
South	1.26 (0.55–2.86)	0.58	0.69 (0.44–1.10)	0.12	0.91 (0.58–1.43)	0.69	0.61 (0.33–1.10)	0.10	0.76 (0.49–1.17)	0.21
West	0.77 (0.22–2.68)	0.69	0.51 (0.23–1.18)	0.09	0.66 (0.32–1.37)	0.26	0.53 (0.19–1.45)	0.22	0.67 (0.33–1.36)	0.26

OR (95% CI) shows the odds ratio and 95% confidence intervals. * Denotes statistical significance (*p* < 0.05). ^a^ Models adjusted for concern about contracting COVID-19. ^b^ Males coded as the reference category. ^c^ Race could not be included in the model about concern for contracting COVID-19 due to the limited number of cases across categories.

**Table 5:** Logistic regression models describing associations between demographic characteristics and respondent’s willingness to have their pets tested for SARS-CoV-2.

	Free Test for SARS-CoV-2 for Pets		Paid Test for SARS-CoV-2 for Pets	
Demographic Characteristics	OR (95% CI)	*p*	OR (95% CI)	*p*
Sex ^a^	0.91 (0.58–1.43)	0.68	1.18 (0.72–1.92)	0.51
Age				
18–29	Reference		Reference	
30–39	0.55 (0.37–0.81)	0.002 *	1.02 (0.68–1.54)	0.91
40–49	0.73 (0.48–1.10)	0.14	1.20 (0.77–1.89)	0.42
50–59	0.54 (0.37–0.80)	0.002 *	1.08 (0.71–1.63)	0.73
60+	0.67 (0.45–1.00)	0.05 *	1.27 (0.83–1.93)	0.27
Race				
Caucasian	Reference		Reference	
African American	1.66 (0.80–3.46)	0.18	0.69 (0.29–1.65)	0.40
Asian	2.00 (0.75–5.31)	0.17	1.60 (0.68–3.78)	0.29
Ethnicity				
Hispanic/Latino/Spanish	0.50 (0.23–1.08)	0.08	0.52 (0.21–1.34)	0.17
Education				
High school or less	Reference		Reference	
Undergraduate	0.76 (0.51–1.13)	0.18	0.87 (0.56–1.34)	0.52
Postgraduate	0.74 (0.48–1.14)	0.18	0.94 (0.60–1.49)	0.80
Household income				
<$30,000	Reference		Reference	
$30,000–$49,999	0.96 (0.57–1.62)	0.88	2.27 (1.26–4.11)	0.01 *
$50,000–$99,999	0.85 (0.53–1.34)	0.48	1.66 (0.97–2.85)	0.07
$100,000–$349,999	0.90 (0.56–1.44)	0.66	1.95 (1.13–3.36)	0.01 *
>$350,000	0.78 (0.37–1.65)	0.52	3.18 (1.43–7.08)	0.01 *
Number of people in household				
1–2 people	Reference		Reference	
3–4 people	0.93 (0.70–1.23)	0.61	0.83 (0.61–1.12)	0.22
5+ people	0.57 (0.32–1.02)	0.06	0.66 (0.35–1.24)	0.19
US region				
Northeast	Reference		Reference	
Midwest	0.44 (0.17–1.13)	0.09	0.78 (0.29–2.09)	0.62
South	0.80 (0.52–1.25)	0.33	0.97 (0.61–1.55)	0.90
West	1.00 (0.49–2.02)	1.00	0.70 (0.32–1.51)	0.36
Concern regarding COVID-19				
Not concerned	Reference		Reference	
Somewhat concerned	2.50 (1.45–4.29)	0.001 *	2.42 (1.20–4.86)	0.01 *
Moderately concerned	3.47 (2.03–5.93)	<0.001 *	3.00 (1.51–5.97)	0.002 *
Fairly concerned	5.55 (3.22–9.59)	<0.001 *	4.32 (2.18–8.59)	<0.001 *
Very concerned	6.68 (3.71–12.04)	<0.001 *	7.37 (3.61–15.03)	<0.001 *

OR (95% CI) shows the odds ratio and 95% confidence intervals. * Denotes statistical significance (*p* < 0.05); ^a^ Males were coded as the reference category. Models were adjusted for respondent’s concern about contracting COVID-19.

## Data Availability

The data presented in this study are available on request from the corresponding author. The data are not publicly available due to ethical restrictions.

## Supplementary Materials

The following supporting information can be downloaded at: https://www.mdpi.com/article/10.3390/vetsci9050195/s1, Supplementary Material: Survey Questions.
