# Supplementary material for: Pet Owners’ Perceptions of COVID-19, Zoonotic Disease, and Veterinary Medicine: The Impact of Demographic Characteristics
Source: Vet Sci. 2022 Apr 19;9(5):195. doi: 10.3390/vetsci9050195 (PMC9143664; doi:10.3390/vetsci9050195)
Supplement: Supplementary file 1 [file vetsci-09-00195-s001.zip › vetsci-1658983-supplementary.pdf]

Survey Questions:

1. Zipcode (enter numeric value)
2. Age
    - 2.1. 18-29
    - 2.2. 30-39
    - 2.3. 40-49
    - 2.4. 50-59
    - 2.5. 60-69
    - 2.6. 70+
3. Sex assigned at birth
    - 3.1. Female
    - 3.2. Male
    - 3.3. Intersex
    - 3.4. No Answer
4. Gender Identity
    - 4.1. Man
    - 4.2. Woman
    - 4.3. Non-binary
    - 4.4. Other, not-listed
    - 4.5. No Answer
5. Are you Hispanic, Latino, or of Spanish origin?
    - 5.1. Yes
    - 5.2. No
6. Race/Ethnicity
    - 6.1. White
    - 6.2. Black or African American
    - 6.3. American Indian or Alaska Native
    - 6.4. Asian
    - 6.5. Native Hawaiian and Pacific Islander
    - 6.6. Other, not-listed
    - 6.7. No Answer
7. Annual household income before taxes in 2019
    - 7.1. Less than $30,000
    - 7.2. $30,000 - $49,999
    - 7.3. $50,000 - $99,999
    - 7.4. $100,000 - $349,999
    - 7.5. More than $350,000
8. How many people are staying in this house, apartment, or mobile home? (enter numeric value)
    Count everyone living and sleeping in your home most of the time, including young children, roommates, and friends and family members who are living with you, even temporarily
9. Education level (highest level completed)
    - 9.1. No degree earned
    - 9.2. High school diploma or GED
    - 9.3. Associate’s

9.4. Bachelor’s
9.5. Masters
9.6. Doctorate

10. Do you have any pets?
    10.1. Yes
        10.1.1. Dog
            10.1.1.1. How many
        10.1.2. Cat
            10.1.2.1. How many
        10.1.3. Other (enter text value - please list how many other pets and of what kind)
    10.2. No
11. Are your pets up to date on vaccinations?
    11.1. Yes
    11.2. No
12. Have you been able to access veterinary care?
    12.1. Yes
    12.2. No
    12.3. N/A
13. In general, how difficult is it for you to access veterinary care currently?
    13.1. Scale of 1-5 (extremely difficult - somewhat difficult – neither easy nor difficult – somewhat easy - extremely easy)
        13.1.1. What has prevented you from accessing veterinary care? *Select all that apply*
            13.1.1.1. Cost of services/ability to pay
            13.1.1.2. Distance/ inadequate transportation
            13.1.1.3. Language/ communication barriers
            13.1.1.4. Other (enter text value)
14. How affordable is veterinary care for you at this time?
    14.1. Scale of 1-5 (extremely unaffordable - somewhat unaffordable – neither unaffordable nor affordable – somewhat affordable – very affordable)
15. Do you have pet insurance?
    15.1. Yes
    15.2. No
16. In an emergency situation, how much do you think you would be willing to spend on veterinary care for your pet? (enter numeric value)
17. Have you heard the term zoonotic disease before?
    17.1. Yes
    17.2. No

Zoonotic disease definition: diseases spread between animals and people. Examples of zoonotic diseases include rabies, west nile virus, swine flu, ringworm, etc.

18. Prior to this survey, did you know what a zoonotic disease was?
    18.1. Yes
    18.2. No
19. How concerned are you about your pets getting zoonotic diseases?

19.1. Scale of 1-5 (not concerned – somewhat concerned - moderately concerned – fairly concerned - very concerned)

20. How concerned are you about giving zoonotic diseases to your pets?
    20.1. Scale of 1-5 (not concerned – somewhat concerned - moderately concerned – fairly concerned - very concerned)
21. How concerned are you about getting zoonotic diseases from your pets?
    21.1. Scale of 1-5 (not concerned – somewhat concerned - moderately concerned – fairly concerned - very concerned)
22. In general, has your access to veterinary care changed since the beginning of the COVID-19 pandemic?
    22.1. Scale of 1-5 (much more difficult – somewhat more difficult - no change – somewhat easier - much easier)
        22.1.1. What changed about your access to veterinary care? *Select all that apply*
            22.1.1.1. Cost of services/ability to pay
            22.1.1.2. Distance/ inadequate transportation
            22.1.1.3. Language/ communication
            22.1.1.4. Other (enter text value)

COVID-19 or Coronavirus is a suspected zoonotic disease, though the American Veterinary Medical Association (AVMA) states on June 11, 2020 that "there is little to no evidence that domestic animals are easily infected with SARS-CoV-2 [the virus causing the disease] under natural conditions and no evidence to date that they transmit the virus to people. The primary mode of transmission of COVID-19 in humans is person-to-person spread."

23. How concerned are you about getting COVID-19?
    23.1. Scale of 1-5 (not concerned – somewhat concerned - moderately concerned – fairly concerned - very concerned)
    23.2. N/A
24. How concerned are you about your pets getting COVID-19
    24.1. Scale of 1-5 (not concerned – somewhat concerned - moderately concerned – fairly concerned - very concerned)
25. How concerned are you about transmitting COVID-19 to your pets?
    25.1. Scale of 1-5 (not concerned – somewhat concerned - moderately concerned – fairly concerned - very concerned)
26. How concerned are you about getting COVID-19 from your pets?
    26.1. Scale of 1-5 (not concerned – somewhat concerned - moderately concerned – fairly concerned - very concerned)
27. How likely are you to ask a physician about zoonotic diseases?
    27.1. Scale of 1-5 (extremely unlikely – somewhat unlikely – neither likely nor unlikely – somewhat likely - extremely likely)
28. How likely are you to ask a veterinarian about zoonotic diseases?
    28.1. Scale of 1-5 (extremely unlikely – somewhat unlikely – neither likely nor unlikely – somewhat likely - extremely likely)
29. Has a physician ever talked to you about zoonotic diseases?
    29.1. Yes

29.2. No

30. Has a veterinarian ever talked to you about zoonotic diseases?
    30.1. Yes
    30.2. No

The American Veterinary Medical Association (AVMA) states as of May 26, 2020 that "routine testing of animals for COVID-19 is not recommended by the AVMA, CDC, USDA, American Association of Veterinary Laboratory Diagnosticians (AAVLD), National Association of State Public Health Veterinarians (NASPHV), or the National Assembly of State Animal Health Officials." *https://www.avma.org/resources-tools/animal-health-and-welfare/covid-19/testing-animals-sars-cov-2*

31. If their recommendations were to change, how likely would you be to get your pet tested for COVID-19 if the test was free?
    31.1. Scale of 1-5 (extremely unlikely – somewhat unlikely – neither likely nor unlikely – somewhat likely - extremely likely)
32. If their recommendations were to change, how likely would you be to get your pet tested for COVID-19 if you had to pay for the test?
    32.1. Scale of 1-5 (extremely unlikely – somewhat unlikely – neither likely nor unlikely – somewhat likely - extremely likely)
33. If you had to pay to get your pet tested for COVID-19, how much would you be willing to pay? *(In US dollars)* (enter numeric value)
34. How comfortable are you with having a COVID-19 positive pet in your home?
    34.1. Scale of 1-5 (extremely uncomfortable – somewhat uncomfortable – neither comfortable nor uncomfortable – somewhat comfortable - extremely comfortable)
